# Taiwan’s ecological footprint and overshoot day

**DOI:** 10.1038/s41598-021-94540-7

**Published:** 2021-07-23

**Authors:** Yung-Jaan Lee, Lei Chai, Po-Shu Wu

**Affiliations:** Chung-Hua Institution for Economic Research, Taipei, Taiwan

**Keywords:** Environmental sciences, Environmental social sciences

## Abstract

This study examines Taiwan’s ecological footprint (EF) and its Overshoot Day from 2000 to 2018. The latest EF calculation method is used to determine the conversion rates and equivalent factors of bioproductive lands in each year to establish a database of Taiwan’s EF in that period. The results reveal that Taiwan’s EF was 7.69 gha/person in 2000, dropping steadily to 6.46 gha/person in 2018. Taiwan’s carbon footprint accounted for about 61% of Taiwan’s total EF, slightly higher than the world average (60%). The carbon footprint as a proportion of the total EF has been increasing annually. This study adopts social communication tools, such as the overshoot day and the earth clock, to promote sustainable development goals and climate change policy initiatives. Global Footprint Network (GFN) updates the overshoot day of each country in its database yearly, based on each country’s EF and biocapacity. Since Taiwan is not included in GFN, this study adopts the same method and finds out that Taiwan's Overshoot Day in 2018 was March 14th, meaning that on March 14th, 2018, Taiwan exhausted all of the biological resources that its bioproductive lands can regenerate in the year. If the global population lived like Taiwanese, four Earths would be required to provide the resources used. This result not only reflects the consumption of natural resources in Taiwan, but also indicates that Taiwan should focus on sustainable development and reduce that consumption.

## Introduction

Ecological footprint (EF) is a widely used sustainability indicator^1–4^ to measure ecological demands of human^5^ and judge human pressures on the ecosystem^6^. As Ahmed et al.^5^ point out that human demands for ecological resources have exceeded the biocapacity and led to overshoot, indicating that the use of the Earth’s resources is higher than its ability to regenerate them^2,8^. It is thus suggested that it takes more than 1 year and a half to regenerate the Earth’s resources that human consume in a year^7^. The Global Footprint Network (GFN) regularly publishes studies that provide the EFs of 234 nations and territories around the world and updating calculation methods and related parameters^8^. As suggested, the acceleration of urbanization and industrialization has posed new ecological security risks to metropolitan areas in low-income countries^9^.

Conversely, COVID-19 swept the world in 2020 and global pandemic prevention and control measures have reduced human economic activities. According to the latest research from GFN, the COVID-19 pandemic has resulted in a sharp fall in the global EF in 2020^10^. The data reveal that the footprint associated with forest products has fallen by 8.4%, that associated with food remained flat (even as the population grew by 1%)^11^, and the carbon footprint fell significantly by 14.5%: this was the first year in which the carbon footprint decreased since the global financial crisis in 2008.

Taiwan’s performance in responding to and protecting against the COVID-19 pandemic in 2020 has been relatively effective^12,13^. The relationship between the pandemic and the sustainability agenda should now be carefully reviewed^14–16^. Taiwan’s EF over recent years can be calculated to help to examine this relationship. Since Taiwan is not included in the GFN, few relevant research results and data on Taiwan are available. For a long time, Taiwan’s EF has for years been calculated by the Forestry Bureau of the Council of Agriculture to generate a database of the country’s EF over time^17^.

Taiwan’s EF data are available for 2000–2011, but no systematic study of Taiwan's EFs has been conducted since 2012^18^. Accordingly, the purpose of this study is to update and calculate Taiwan’s EFs from 2000 to 2018. The latest methods are used to re-calculate Taiwan’s EFs from 2000 to 2011 and Taiwan’s EFs from 2012 to 2018 are obtained. The database of Taiwan’s EFs thus obtained enables the long-term trend of Taiwan’s EF to be tracked.

As GFN and other organizations have tracked the global EF for a long time, the EF has increasingly become an effective indicator of sustainability. Lin et al.^2^ collated EF with other development indicators, such as the Human Development Index (HDI). They pointed out^2^ that the relationship between national EF and the HDI is convex: low EF is associated with low HDI and high EF is associated with high HDI. Does this fact imply that almost no country can have a high HDI within the range of biological tolerance? Or may a high level of human development come at the cost of ecological overshoot?

GFN and other organizations have developed other concepts for social communication. These include Overshoot Day, on which a region or country begins to use more than its own natural resources. After overshoot day, the region or country is appropriating resources that will be needed in the future. Therefore, an earlier overshoot day corresponds to less sustainable consumption and life^10^.

## Methods and materials

EF is a sustainability monitoring indicator that has been widely used by the international community in recent years. In 1994, Mathis Wackernagel and his Ph.D. advisor William Rees, a Canadian ecological economist, proposed the concept of EF^19^. Its innovation is in shifting our understanding of sustainable development from narrative to quantitative^20^. Wackernagel et al*.*^21^ first calculated the EF of nations. Subsequently, Lin et al.^8^ published the latest guidebook of GFN. The guidebook, which has been updated periodically, associated EF with six types of bioproductive land (cropland, grazing Land, forest products, fishing grounds, built-up land, carbon footprint), establishing an important tool for measuring progress toward sustainable development. EF can directly or indirectly quantify the relationship between human society and the natural environment. It can also serve as an indicator of measuring the balance between human society and ecosystem services. It reflects the impact of human activities on the natural environment and can be used to calculate the natural resources that are made available to humans via ecosystem services^3^.

In practice, only the basic services for human activities have to be calculated, and double-calculation must be avoided. The EF account is based on a calculation of the per capita consumption (Ci) of six consumption items (i) from statistical data. To convert the six consumption values into a single value, the per capita consumption (Ci) is divided by the average productivity (Pi) of each item (i), and the six values thus obtained are summed to yield the EF per capita:

1 $${\text{EF}}\;\left( {{\text{gha}}/{\text{person}}} \right) = {\text{Ci}}/{\text{Pi}}$$

where Ci (Per capita consumption): For a consumption item, the regional or national total weight is divided by the total population, and the result given in units of weight, usually kilograms or tons. Pi (average productivity): The production volume per unit area (conversion value) of a region or country. Production volume is usually expressed in metric tons, and area is often expressed in hectares. Unified value: The bioproductive land area can be simplified to a global hectare (gha) value.

In the calculation process, the EF must be corrected for trade and equivalent productivity to reflect (1) frequent global economic and trading exchanges, including the import and export of consumer goods, (2) the fact that the productivity of land with respect to different consumer products is not always the same. For example, arable land is highly intensively managed and therefore has high productivity. Grassland is highly extensively managed and therefore has low productivity. Therefore, an "equivalent correction" of the data is required, and it is carried out using the "equivalent factor" (EQF), which is calculated as follows^8^.

### EF of cropland, grazing land, fishing grounds and forest products (gha)

2 $${\text{EF}}\;\left( {\text{i}} \right) = {\text{Total consumption}}\;\left( {\text{i}} \right)/{\text{Population}}/{\text{Bioproductivity}}\;\left( {\text{i}} \right) \times {\text{EQF}}\;\left( {\text{i}} \right)$$

The total consumption is the domestic production plus the imported amount minus the exported amount, in tons; the bioproductivity and equivalent factors of different consumption items are different.

### EF of built-up land (gha)

3 $${\text{EF}} = {\text{Built-up land area}} \times {\text{Bioproductivity of cropland}}/{\text{Population}} \times {\text{EQF}}$$

The unit of built-up area is hectare. Since built-up land is assumed to have been converted from productive cropland, calculation of the EF of built-up land assumes that the productivity and equivalent factors of the built-up land are the same as those of the cropland.

### Carbon footprint (gha)

4 $${\text{EF}} = {\text{Carbon emission per capita}} \times (1 - {\text{Ocean absorption rate}})/{\text{Carbon fixation rate}} \times {\text{EQF}}$$

Carbon emission per capita is given in units of ton(s)/person; the ocean absorption rate is the proportion of the carbon dioxide that is absorbed by the ocean that was emitted by human activities; the carbon fixation rate is given in units of ton(s)/ha and represents the amount of carbon that can be absorbed per hectare of forest land.

The EF calculation consists of two parts. The first part determines the EF on the consumption/demand side; the second determines the biocapacity on the production/supply side. These two values are evaluated on a particular scale (global, national or local) and are used in the basic formula for environmental sustainability, which is Ecological Footprint (EF) − Biocapacity (BC) = Ecological Deficit (ED)^17^. ED reflects the over-utilization of natural resources. A larger deficit represents greater severe over-utilization. According to the relevant statistics, the world shifted from an ecological surplus to an ecological deficit around 1970^2^, when the total amount of resources consumed in human activities exceeded the environmental assimilation capacity (also known as the self-purification capacity) of the earth. Accordingly, since 1970, we have been consuming natural resources that should be available to future generations and increasing annual environmental impact.

Lin et al.^2^ reviewed global EF research from 2012 to 2018, focusing on the changes in research methods, calculation formulas, and sources of data for determining EF during that period. The most important influence of the changes on the EF account was the update to the footprint calculation formula that was proposed by Mancini et al.^22^.

The main purpose of this study is to calculate Taiwan's EF and overshoot day. The updated carbon footprint calculation formula has had a significant impact on Taiwan's EF research—most significantly by adjusting the carbon sequestration rate. The formula for the carbon sequestration rate is as follows.

$${\text{Y}}_{{\text{c}}} = {\text{AFCS}}/{\text{CCR}}$$

where Y_c_: annual average carbon sequestration rate per hectare of forest land. AFCS: annual average forest carbon sequestration. CCR: carbon and carbon dioxide conversion rate, 0.27^22^.

In calculations of the carbon footprint before 2016, the values ​​of AFCS were 0.97 t C ha^−1^ yr^−1^ (t: tons; C: carbon; ha: hectare; yr: year)^22^. In 2015, Mancini et al.^22^ revised the AFCS to 0.73 t C ha^−1^ yr^−1^ to reflect more realistic carbon dioxide emissions and forest degeneration, and revised the carbon sequestration rate (Y_c_) from 3.4 to 2.7. In the carbon footprint calculation formula above, the carbon sequestration rate (YC) is in the denominator so a decrease in the carbon sequestration rate increases the overall carbon footprint. Since 2016, the GFN guidebook has used the carbon footprint calculation method as revised by Mancini et al.^22^. Lin et al.^2^ used this same new carbon footprint calculation method to recalculate the global EF over multiple years.

## Results

The results of this study are presented in several sections. The first section reviews Taiwan’s first-wave study of EF from 2000 to 2011, and recalculates it using the latest research methods. In the second section, latest research methods are used to update Taiwan’s EF for 2012 to 2018; the method for classifying bioproductive land that was adopted by Lin et al.^8^ is used. In the third section, Taiwan's biocapacity and ecological deficit are calculated, and a comprehensive analysis of the ecological deficit over recent years is presented. The final section responds to the review of EF research and social communication by Lin et al.^2^, calculating Taiwan’s overshoot days from 2000 to 2018. Overshoot day is one of the indicators of Taiwan’s sustainable development, and it is used herein in the hope of communicating information related to sustainable development to members of society.

### Review of EF studies from 2000 to 2011

Taiwan’s EF was calculated for the first time in 1998 using the method of Wackernagel and Rees^23^, which divides bioproductive land into six major types (cropland, grazing land, fishing grounds, forest products, energy land, and built-up land). In 1996, Taiwan’s EF was 4.67 hectares/person. However, this value was not corrected the equivalent factor (EQF), and so was a gross underestimate. In 2005, Taiwan’s Council of Agriculture (COA) commissioned Lee and his colleagues to incorporate the equivalent factor into the calculation of EF. They found that EF increased from 5.07 gha/person in 1994 to 5.14 gha/person in 2003^24^.

Since the global warming that is caused by increasing greenhouse gas emissions threatens global sustainable development, to reflect the increase in carbon emissions that is increasing the EF value, GFN replaced the energy land with carbon footprint. Lee^24^ then recalculated the EF of Taiwan for 2004, and found it to have increased by a factor of approximately 1.3 from 5.14 to 6.72 gha (of which the carbon footprint accounted for 2.23 gha).

Wang et al.^25^ calculated Taiwan’s EF for 2007 as 6.54 gha. The most important changes in the EF calculation concern the built-up land and the energy land. First, since obtaining hydropower data is difficult, the built-up land footprint is calculated only using the areas of buildings in the built-up areas. Second, the energy land footprint was originally divided into “the consumer goods footprint” and "carbon footprint" but consumer goods are complicated and difficult to handle statistically. Therefore, items related to consumer goods are excluded from the calculation of energy land and only carbon emissions are considered. To respond to global trends in EF calculation and to continue to track Taiwan's EF, Lee and Peng^17^ calculated Taiwan's EF for 2011, and found it to be 9.43 gha/person, of which the carbon footprint was as high as 5.94 gha/person.

International EF research trends and the latest calculation methods are considered here. Methods of calculation of Taiwan's EF and the results obtained using them for 2000 to 2011 are reviewed. The carbon sequestration rate (Y_c_) that was applied in the carbon footprint calculation formula of Mancini et al.^22^ before 2016 was 3.4, but the carbon sequestration rate (Y_c_) that was used by Lee and Peng^17^ to calculate the carbon footprint of Taiwan for 2000–2011 was 1.8. Substituting the corrected carbon sequestration rate into the carbon footprint calculation formula yields,

5 $${\text{EF}} = {\text{Carbon emission per capita}} \times (1 - {\text{Ocean absorption rate}}) \times {\text{EQF}}/{\text{Y}}_{{\text{c}}}$$

The unit of carbon emission per capita is ton(s)/person. The ocean absorption rate that was calculated in 2014 was 1/4^17^. The carbon sequestration rate was 3.4^20^ and 1.8^17^. This change to the different carbon sequestration rate nearly doubled carbon footprint, increasing Taiwan’s overall EF by 2–3 global hectares/person (Table 1).

**Table 1:** Impact of carbon sequestration rate on EF from 2000 to 2011 (unit: gha).

EF	2000	2001	2002	2003	2004	2005
Yc = 1.8	10.47	9.39	9.76	9.98	8.95	9.64
Yc = 3.4	7.69	6.91	7.20	7.31	6.20	6.85

EF	2006	2007	2008	2009	2010	2011
Yc = 1.8	9.49	9.63	9.12	8.80	9.53	9.55
Yc = 3.4	6.63	6.73	6.35	6.19	6.73	6.70

The difference in the carbon footprints that were calculated using different carbon sequestration rates explains the value difference in the EF data from 2000 to 2011. In this study, the original data are adjusted and Taiwan’s EF data from 2000 to 2011 are revised accordingly. Carbon footprint and EF are calculated using methods adopted in international research. The revised footprints of all types of bioproductive land and the total EF in Taiwan from 2000 to 2011 are as follows (Table 2).

**Table 2:** Taiwan’s EF from 2001 to 2011 (unit: gha).

Years	Cropland	Grazing land	Fishing grounds	Forest products	Built-up land	Carbon footprint	EF
2000	4.44	0.04	0.10	0.42	0.16	2.53	7.69
2001	3.69	0.03	0.17	0.33	0.13	2.56	6.91
2002	3.86	0.04	0.17	0.37	0.12	2.64	7.20
2003	3.80	0.04	0.18	0.42	0.13	2.74	7.31
2004	2.62	0.03	0.15	0.45	0.13	2.82	6.20
2005	3.18	0.04	0.15	0.44	0.16	2.88	6.85
2006	2.99	0.04	0.15	0.34	0.15	2.96	6.63
2007	3.02	0.03	0.17	0.38	0.15	2.98	6.73
2008	2.77	0.03	0.16	0.38	0.16	2.85	6.35
2009	2.95	0.03	0.14	0.22	0.16	2.69	6.19
2010	3.10	0.03	0.14	0.39	0.19	2.88	6.73
2011	3.01	0.03	0.16	0.38	0.19	2.93	6.70

### Calculation of EF from 2012 to 2018

The classification method of Lin et al.^8^ is used to divide the EF into six components, corresponding to cropland, grazing land, fishing grounds, forest product, built-up land, and carbon footprint.

1. Footprints of Cropland, Grazing Land, Fishing Grounds, and Forest Products

The footprints of cropland, grazing land, fishing grounds, and forest products, must be calculated separately for each consumption item (i). The footprints of all consumption items are then summed. Cropland footprints are components associated with cereals, potatoes, sugar and honey, seeds and oilseeds, and fruits (including vegetables); grazing land footprints are components associated with meat and oils; fishing ground footprints are associated only with aquatic products; forest product footprints are associated only with timber. The relevant equation is as follows.

6 $${\text{EF}}\;\left( {\text{i}} \right) = {\text{Total consumption}}\;\left( {\text{i}} \right)/{\text{Population}}/{\text{Bioproductivity}}\;\left( {\text{i}} \right) \times {\text{EQF}}\;\left( {\text{i}} \right)$$

Total consumption must be adjusted for international trade, by calculating domestic production, adding imports and subtracting exports. Population here is the total population of Taiwan. Bioproductivity is the global bioproductivity (conversion rate) of a certain consumption item, which is the total production of consumption item (i) in the world divided by the area that is used to cultivate ​​that item (i). Different types of bioproductive land have different equivalence factors.

2. Footprint of Built-up Land

The EF calculation assumes that built-up land was converted from cropland^21^. Therefore, all built-up land is assumed to have the same bioproductivity as cropland. Therefore, the bioproductivity and equivalent factors of built-up land are the same as those of cropland. Hence, only the built-up land area has to be calculated to obtain the built-up land footprint.

7 $${\text{EF}} = {\text{Built-up land area}} \times {\text{Bioproductivity of cropland}}/{\text{Population}} \times {\text{EQF}}$$

The area of ​​built-up land is expressed in hectares and this land can be divided into urban land and non-urban land. Population is the total population of Taiwan. The calculation of EF assumes that the bioproductivity and equivalent factor of built-up land are the same as those of cropland.

3. Carbon Footprint

The carbon footprint represents the area of land that is required to absorb the CO_2_ that is emitted by human activities. In calculating EF, the carbon footprint must be calculated independently to address the carbon emissions that are caused by human activities. However, in calculating biocapacity, because the carbon footprint shares the same land areas with the forest land footprint the calculation must not be double-counted. The carbon footprint is calculated as follows.

8 $${\text{EF}} = {\text{Carbon emission per capita}} \times (1 - {\text{Ocean absorption rate}})/{\text{Carbon sequestration rate}} \times {\text{EQF}}$$

The unit of per capita carbon emission is ton(s)/person. The ocean absorption rate is the proportion of the total amount of CO_2_ that is emitted in human activities that is absorbed by the ocean. Lin et al.^8^ provided the most recently determined ocean absorption rate of 0.281. The carbon sequestration rate is expressed in ton(s)/hectare, and it represents the amount of carbon that can be fixed per hectare of forest land. In this study, the updated Y_c_ = 2.7^22^ is used in calculations. Table 3 presents the calculated EF for Taiwan from 2012 to 2018.

**Table 3:** Footprints of six types of bioproductive land from 2012 to 2018 (unit: gha).

Land types	2012	2013	2014	2015	2016	2017	2018
Cropland	2.05	1.79	1.88	1.90	1.80	1.85	1.81
Grazing land	0.10	0.10	0.11	0.11	0.12	0.12	0.12
Fishing grounds	0.21	0.18	0.17	0.16	0.18	0.18	0.18
Forest products	0.35	0.32	0.34	0.32	0.27	0.25	0.27
Built-up land	0.19	0.19	0.19	0.19	0.19	0.19	0.19
Carbon footprint	3.71	3.71	3.79	3.79	3.84	3.93	3.89
EF total	6.61	6.30	6.48	6.47	6.40	6.51	6.46

**Figure 1:**
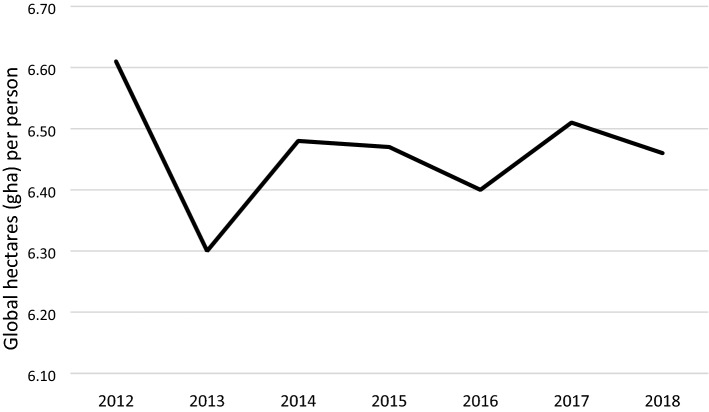
Taiwan's EF from 2012 to 2018.

From 2012 to 2018, Taiwan's EF slowly declined, although the decline in 2013 was particularly large because of a decline in the cropland footprint, including low domestic production and exports^26^. According to statistics from the Central Weather Bureau^27^ of the Ministry of Transportation and Communications, warnings for nine typhoons were issued in 2013—the largest annual number in the last decade. Therefore, the number of typhoons might have been responsible for the low cropland production in that year, affecting the cropland footprint in that year. The EF in 2013 was significantly lower than in the other years of interest (Fig. 1).

**Figure 2:**
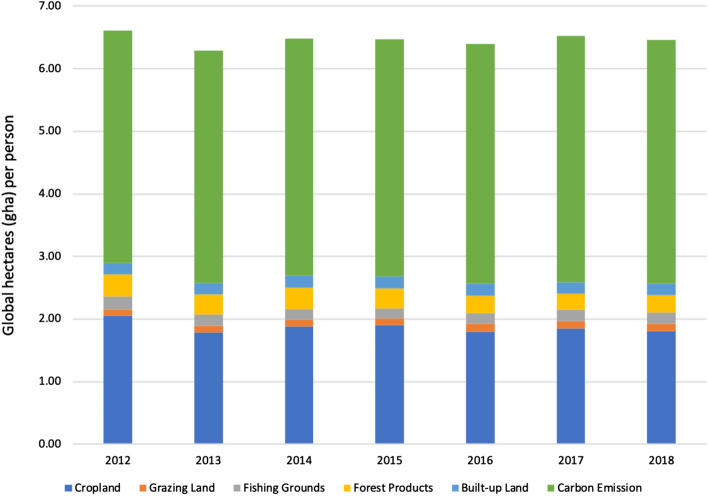
Changes in the footprints of six types of bioproductive land from 2012 to 2018.

Taiwan’s overall EF trends from 2012 to 2018 and the various footprints (Fig. 2) reveal that carbon footprint and cropland footprint account for a high proportion of the overall EF. From 2012 to 2018, Taiwan’s EF had been slowly decreasing. The cropland footprint declined considerably while the carbon footprint continuously increased. Despite the global emphasis on carbon emissions and their reduction, Taiwan’s carbon footprint increased throughout the period of interest. Carbon reduction is a critical goal of Taiwan’s future energy and environmental policies.

The carbon footprint is the main contributor to Taiwan's EF; it is followed by the cropland footprint. The cropland footprint represents about 20% of the total EF, while the carbon footprint accounts for about 61%. The other four footprints represented small fractions of the total EF and did not change significantly.

### Ecological deficit

This section compares Taiwan’s EF with global EF. It will first explain the calculation of biocapacity, and then consider EF to obtain Taiwan’s ecological deficit. Since EF per capita is considered, it should be divided by the total population of Taiwan to obtain the biocapacity per capita for Taiwan, as follows.

9 $${\text{Bioproductive land area}} \times {\text{Yield factor}} \times {\text{EQF}}/{\text{Population}}$$

Bioproductive land area is measured in hectares. The yield factor compares the biological productivity of a specific consumption item (i) in a certain area with the global bioproductivity thereof as a ratio. In this study, the yield factor is Taiwan’s bioproductivity (conversion rate) divided by the global bioproductivity (global conversion rate). The equivalent factor and total population are as in the calculation of EF in the preceding section.

**Figure 3:**
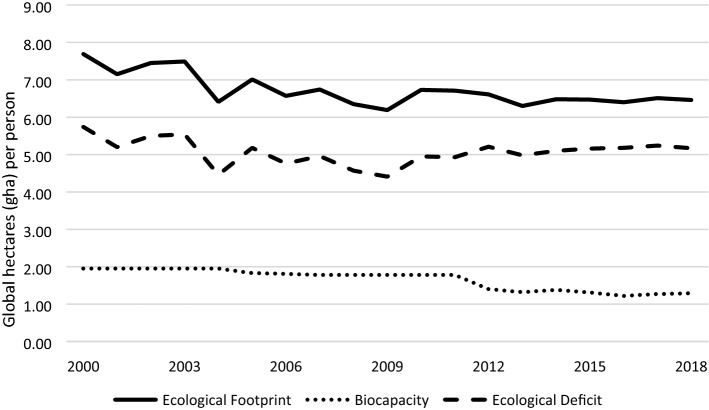
Changes in Taiwan's EF, biocapacity, and ecological deficit from 2000 to 2018.

Although Taiwan's EF remained roughly stable from 2012 to 2018, its biocapacity continued to decline, causing Taiwan's ecological deficit to rise. Figure 3 presents changes in Taiwan’s EF, biocapacity, and ecological deficit from 2000 to 2018.

### Overshoot day

**Figure 4:**
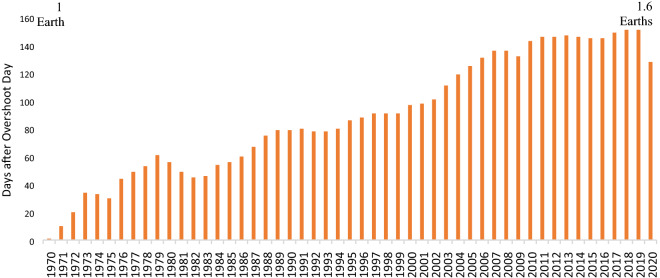
Earth overshoot day from 1970 to 2020. *Source*: Earth overshoot day (2020).

Overshoot day is used to quantify an ecological deficit as it captures how quickly humans are consuming the natural resources that the earth can provide in a year^28^. According to the GFN website, the Earth Overshoot Day in 2020 was August 22, meaning that the world consumed all of the natural resources that the earth could provide to humankind in 2020 by August 22, and through the rest of the year, it appropriated future resources. Since 1970, the global overshoot day has been shifting forward (earlier) every year (Fig. 4), meaning that humans are depleting the earth's natural resources at an increasing rate, and that the over-consumption of natural resources has become increasingly serious. As a result of the pandemic, 2020 is the year with the latest Overshoot Day in the last decade—3 weeks later than in 2019^10^. Therefore, the pandemic has reduced the human consumption of natural resources, giving the earth some respite.

GFN updates the Overshoot Day of each country in its database yearly, based on each country’s EF and biocapacity. Since Taiwan is not included in GFN, this study adopts the same method to calculate Taiwan's Overshoot Day. The calculation divides the biocapacity by EF and multiplies the result by 365 days in a year to yield the number of days by which the ecological limit is exceeded; this number of days is then converted into a date, as shown below.

10 $${\text{Biocapacity}}/{\text{EF}} \times {365} = {\text{Days}}$$

**Figure 5:**
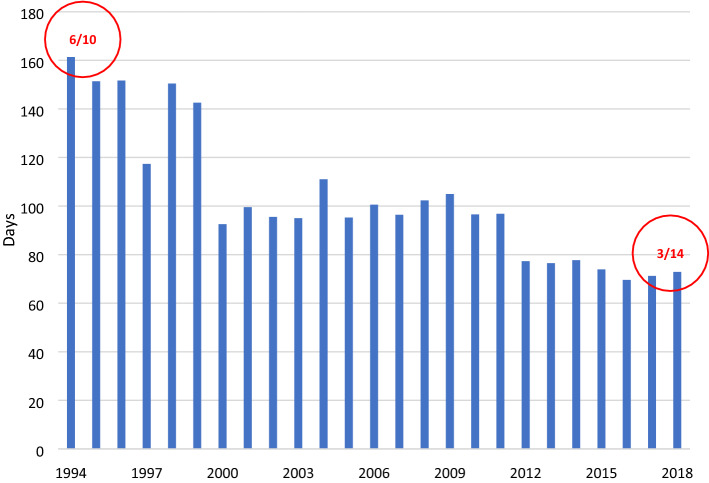
Taiwan’s overshoot day from 1994 to 2018.

The Overshoot Day can be used to determine whether Taiwan is moving toward sustainable development. According to Fig. 5, the ecological Overshoot Day in Taiwan shifter earlier every year from 1994 to 2018. Taiwan’s Overshoot Day in 1994 was June 10, and in 2018 was March 14, nearly 3 months sooner, reflecting the fact that Taiwan is moving not toward but away from sustainability, continuing to appropriate the natural resources of other regions or future generations.

GFN and other, related organizations have designed websites and mobile apps that facilitate the calculation of personal EF and personal overshoot days, so that individuals can quickly determine how quickly the earth’s natural resources will be depleted were the world’s population to share their lifestyle. For example, if the world’s population lived like Americans, humans would need five earths to provide the consumed natural resources^28^. The US is used as a benchmark herein. The U.S.’ EF in 2018 was about 8.1 gha/person, while Taiwan’s EF in 2018 was 6.46 gha/person. Similarly, if the world’s population lived like Taiwanese, four earths would be required to support everyone. A comparison of consumption in Taiwan with that in other major countries such as Russia, India, Germany, and neighboring Japan, should motivate Taiwan to focus on sustainable development and reduce its consumption of natural resources.

## Discussion

This research concerns the development of recent EF research by international organizations such as GFN^2,3,22,29^. The latest methods are used herein to calculate Taiwan’s EF from 2000 to 2018. Taiwan’s EF from 2000 to 2011 is reviewed and revised based on the latest global research. Second, Taiwan's EF data to 2018 are updated. Between 2012 and 2018, Taiwan’s EF was stable, but in 2013, fell significantly. From 2012 to 2018, Taiwan’s carbon footprint accounted for about 61% of its overall EF, not only exceeded the international average of 60%, but also reflecting the fact that Taiwan’s carbon emissions and carbon footprint are trending in the opposite direction to the global reduction. Third, although Taiwan’s EF remained stable from 2012 to 2018, Taiwan’s ecological deficit has continued to increase owing to a continuous reduction of biocapacity, reflecting a gradual decline in Taiwan’s natural resources and a worsening unsustainable use of natural resources. Fourth, as one of the indicators of sustainable development, EF must be communicated to the public in a way that they can understand and that motivates them to make favorable changes in their everyday lives. GFN developed the Overshoot Day as a tool for social communication. The calculations in this study indicate that Taiwan's Overshoot Day in 2018 was March 13. On that date, Taiwan had exhausted the natural resources that its bioproductive land can provide, and spent the remaining almost nine months appropriating the resources of other regions or future generations.

Based on this study, reducing Taiwan’s carbon emissions and carbon footprint is a critical goal of future policy. Sustainable development policies in the areas of energy, the environment, the economy and society, and carbon footprint reduction will be crucial. The results of this study provide a reference for future policy formulation and implementation. Both scientific research and policy implementation must be communicated to the public. Taiwan’s Overshoot Day, calculated in this study, is one tool for social communication.

Based on this study, a number of scientific and academic discussions should proceed in the future. These should target the causes of the continuous increase in Taiwan’s ecological deficit, distinguished by climate zone or location; developing EF methods that are suited to local conditions and the characteristics of different countries, climates and geographies; and obtaining first-hand information to make relevant sources of data more complete, and accordingly, Taiwan’s EF will be able to be calculated more completely using various databases and field surveys.

## Data Availability

The datasets used and analyzed during the current study are available from the corresponding author on reasonable request.
